# Pyogenic Granuloma in the Floor of Mouth: A Case Report

**DOI:** 10.31729/jnma.v64i293.9288

**Published:** 2026-01-31

**Authors:** Mahesh Khadka, Rinky Nyachhyon, Shally Raina, Nisha Maharjan, Pragya Poudel, Subhekhysa Gyawali

**Affiliations:** 1 Department of Oral Medicine and Radiology, Nepal Medical College and Teaching hospital, Jorpati, Kathmandu, Nepal; 2 Department of Oral Medicine and Radiology, People’s Dental College and Hospital, Sorakhutte, Kathmandu, Nepal; 3 Department of Oral Pathology, People’s Dental College and Hospital, Sorakhutte, Kathmandu, Nepal

**Keywords:** *floor of mouth*, *pyogenic granuloma*, *solitary lesion*

## Abstract

Pyogenic granuloma is a common benign, non-neoplastic inflammatory hyperplasia of keratinized tissue, typically arising in response to chronic irritation, trauma, or hormonal influences. Recently it has been classified under vascular tumor and termed as a lobular capillary hemangioma by International Society for the Study of Vascular Anomalies. It presents as a solitary, hemorrhagic, often pedunculated, nodules of variable size which typically appears most of the times in the gingiva. This report presents the case of a 43-year-old male with a pyogenic granuloma on the floor of the mouth. This rare location frequently leads to diagnostic uncertainty among clinicians.

## INTRODUCTION

Pyogenic granuloma (PG) is a common benign, non-neoplastic vascular tumor. Poncet and Dor originally reported this lesion in 1897, and Hartzell coined the term “pyogenic granuloma” or “granuloma pyogenicum” in 1904.^1^

It accounts for about 14.4% of the oral mucosal lesion and is among the most common non-neoplastic oral mucosal lesion in Nepal.^2^ PGs occur most commonly in gingiva (75%) followed by lips, buccal mucosa and palate.^3^ However, they can occur in uncommon sites and often create difficulties in clinical diagnosis. Here we are presenting a case of PG in the floor of oral cavity.

## CASE REPORT

A 43-year-old male patient with cancer phobia presented to the Department of Oral Medicine and Radiology at Nepal Medical College and Teaching Hospital (NMCTH), Kathmandu, Nepal, with a painless, persistent intraoral swelling in the floor of the mouth for the past one year. The swelling had an insidious onset, gradually increasing from a small size to its present dimensions over the past year. It was associated with occasional bleeding during tongue movements but not with any difficulty in speech or mastication.

**Figure 1:**
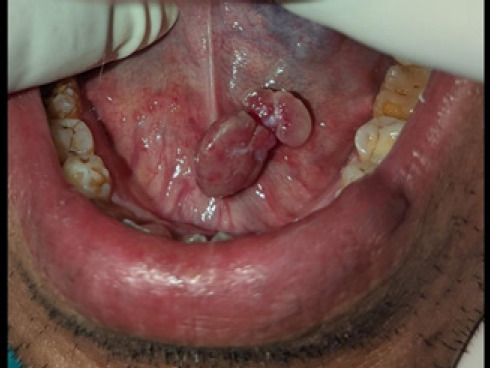
Swelling in floor of the mouth.

On examination, intraorally, two well defined exophytic, lobulated, pinkish pedunculated masses attached to one another with size 1.7 × 1 cm2 at the floor of the mouth near Wharton’s duct with normal surrounding tissue were present which were soft to firm in consistency and non-tender (Figure 1). Salivary flow was normal from the Wharton’s duct which was assessed using manual palpation of the gland.

**Figure 2:**
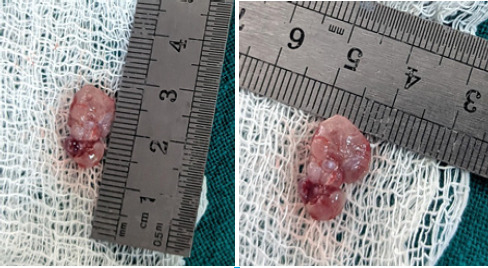
Excised lesion of pyogenic granuloma.

A provisional diagnosis of traumatic fibroma was made and various differential diagnosis of Peripheral Giant Cell Granuloma, Pyogenic Granuloma, Peripheral Ossifying Fibroma and Squamous Cell Carcinoma were given. Excisional biopsy of the lesion from the floor of the mouth was done carefully preserving the Wharton’s duct following which the specimen was sent for histopathological examination. (Figure 2)

**Figure 3:**
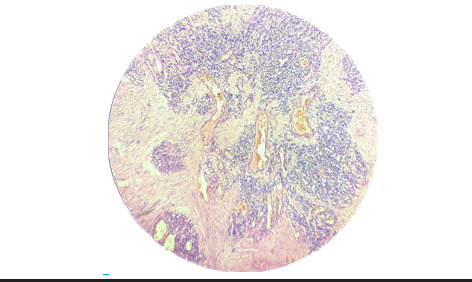
Histopathological sections pyogenic granuloma (10X).

Hematoxylin and eosin-stained (H&E) microscopic examination revealed a cellular mass of connective tissue with intra and perilobular fibrosis over para keratinized stratified squamous epithelium. There was occurrence of small endothelium lined vascular spaces with proliferating endothelial cells and mixed inflammatory cells (Polymorphonuclear leukocytes, Lymphocytes, Plasma cells and mast cells) and areas of hemorrhages also seen along with some focal areas of calcification. The features were suggestive of pyogenic granuloma (involutionary phase) (Figure 3).

Patient was followed up for the next six months and there was no recurrence of the lesion.

## DISCUSSION

Pyogenic Granuloma is considered a misnomer because the lesion does not exhibit granulomatous histology, nor is it linked with suppuration or infectious processes.^4^ Instead, it represents an inflammatory hyperplastic response, usually triggered by local trauma, irritation, or hormonal influences. In recent classifications, the International Society for the Study of Vascular Anomalies has designated it as a lobular capillary hemangioma, placing it within the vascular tumor group.^5^

PG can develop at any age, with a marked female predominance (>2:1 ratio). The maxilla is more frequently involved than the mandible, and the gingiva accounts for the majority of cases (around 75%), followed by the lips, buccal mucosa, and tongue^3^ Unlike these common patterns, our case occurred in a male patient at an uncommon location—the floor of the mouth. A similar presentation of PG at this site have been described in previous international report, where the mass arose from the mucogingival line and reached the floor of the mouth .^6^ However, cases involving the floor of the mouth have not yet been reported in Nepal, and furthermore, the mass arose solely from the floor of the mouth.

The exact mechanism underlying the development of pyogenic granulomas remains uncertain. They are generally considered a reactive lesion arising from a persistent local irritation. Hormonal factors also play a role, particularly during pregnancy, when elevated estrogen levels can lead to the so-called “pregnancy tumor.” These lesions often regress after childbirth as hormone levels return to normal. In the present case, however, no obvious source of irritation or trauma was identified. A possible explanation could be repeated minor, unrecognized injuries occurring during routine daily activities.

Clinically, pyogenic granulomas usually appear as reddish-pink to purplish growths that may be smooth or lobulated and can occur on either a sessile or pedunculated base. Early lesions, being more vascular, often present with a brighter red coloration, whereas older ones tend to appear pink as vascularity decreases. In the present case, the swelling appeared pink, indicating a relatively mature stage of the lesion. Histopathological examination further supported this, revealing features of the involutional phase, including connective tissue with intra- and perilobular fibrosis, along with small endothelial-lined vascular spaces and proliferating endothelial cells.

Sternberg et al. described three distinct stages in the evolution of pyogenic granuloma.^7^ In the early phase, the lesion is composed of a dense cellular stroma with only minimal lumen formation. The capillary phase follows, characterized by lobules containing abundant vascular channels filled with red blood cells. The final involutional phase shows intra- and perilobular fibrosis, representing the healing stage of the lesion. Clinical presentations often reflect these histologic phases: younger lesions are highly vascular and appear red to purple, while older lesions become more fibrotic, giving them a pink appearance.

Clinically, pyogenic granulomas can resemble several other benign soft tissue lesions, including peripheral giant cell granuloma, peripheral ossifying fibroma (POF), and traumatic fibroma, making differentiation challenging without histopathological evaluation. Some reports suggest that POF and traumatic fibroma may represent part of the same disease spectrum as PG. Sridhar R. et al. proposed that long-standing PGs may undergo maturation into POF, indicating a possible continuum between these entities.^8^ Another case report described recurrence of a POF as a PG at the same site within a week of excision, further supporting the close relationship between the two lesions.^9^

Surgical excision remains the primary mode of management, along with removal of the underlying cause. It has a recurrence rate of about 16% following conservative surgical excision.^10^

## CONCLUSION

Pyogenic granulomas may occasionally occur at uncommon or atypical locations; however, the site of the lesion alone should not be relied upon for diagnosis. Histopathological examination can help differentiate it from other benign growths.
